# Operation for Non-union of Fracture of Tibia

**Published:** 1886-07

**Authors:** J. M. Lewis

**Affiliations:** Mexia, Texas


DANIEL'S

### Medical ★ Journal,

PUBLISHED MONTHLY AT

AUSTIN, TEXAS.

Vol. 2.] JULY, 1886. [No. 1.

*"Scribimus indocti, doctique!"*

### Original Articles.

☞ CONTRIBUTED EXCLUSIVELY TO THIS JOURNAL. ☜

*The Articles in this Department are accepted, and published with the understanding that we are not responsible for, nor do we indorse the views and opinions of the writers, by so doing.*

### OPERATION FOR NON-UNION OF FRACTURE OF TIBIA.

*By J. M. Lewis, M. D., Mexia, Texas.*

(For Daniel's Texas Medical Journal.)

MR. N. MOODY, farmer, age about sixty, weight about one hundred and seventy pounds, in good health, was thrown from a wagon and his left leg fractured about six inches below the knee, (both bones broken.)

From the use of badly adjusted splints, the bones failed to unite. Mr. Moody was not able to bear any weight on his leg, and walked by the aid of crutches. The leg could be moved in any direction, and did not cause much pain, the union was ligamentous. This was his condition when I first saw him about two (2) years after the accident.

I explained to him that the only relief was in an operation. With the assistance of Dr. J. H. McCain, who admistered chloroform, I made one straight incision about three (3) inches long, over the false joint and crossed it with one of about the same length; dissecting the flaps back, exposing the place of fracture with a small saw removed the ligamentous tissue and at the same time *freshening* both ends of the bone, (ie. Tibia, for I did not operate on the Fibula.)

After removing all spiculæ of bone, the edges of the wound were brought together with adhesive plaster, and a small point left open for drainage (if any).

The leg was then wrapped in cotton batting, and over this a plaster-of-paris bandage applied; the leg was elevated and a small opening made in the plaster over the wound to allow drainage; and over this cloths wet in solution of carbolic acid.

The wound healed nicely and no discharge. The plaster-of-paris was kept on for about five (5) weeks, when the bandage was removed; union (bony) had taken place, and in a short time he could bear his weight on same, and in three (3) months was walking without the aid of crutch or stick. He is a farmer and does heavy work on the farm. The operation was a complete success.

